# Muscle: an independent contributor to the neuromuscular spinal muscular atrophy disease phenotype

**DOI:** 10.1172/jci.insight.171878

**Published:** 2023-09-22

**Authors:** Narendra N. Jha, Jeong-Ki Kim, Yoon-Ra Her, Umrao R. Monani

**Affiliations:** 1 Department of Neurology,; 2 Center for Motor Neuron Biology and Disease, and; 3 Department of Pathology and Cell Biology, Columbia University Irving Medical Center, New York, New York, USA.

## Abstract

Spinal muscular atrophy (SMA) is a pediatric-onset neuromuscular disorder caused by insufficient survival motor neuron (SMN) protein. SMN restorative therapies are now approved for the treatment of SMA; however, they are not curative, likely due to a combination of imperfect treatment timing, inadequate SMN augmentation, and failure to optimally target relevant organs. Here, we consider the implications of imperfect treatment administration, focusing specifically on outcomes for skeletal muscle. We examine the evidence that muscle plays a contributing role in driving neuromuscular dysfunction in SMA. Next, we discuss how SMN might regulate the health of myofibers and their progenitors. Finally, we speculate on therapeutic outcomes of failing to raise muscle SMN to healthful levels and present strategies to restore function to this tissue to ensure better treatment results.

## Introduction

Spinal muscular atrophy (SMA) is a common, predominantly pediatric neuromuscular disorder that was initially described more than a century ago to be a frequently fatal paralytic condition (type I SMA) (1, 2). Intermediate (type II SMA), mild (type III SMA), and adult-onset forms (type IV SMA) of the disease were subsequently recognized and described (3–8). In the early 1990s, a common locus on the long arm of human chromosome 5 was linked to all four forms of SMA. The description of the SMA-linked chromosome was followed in quick succession by a report revealing survival motor neuron 1 (*SMN1*) as the gene within the locus that was responsible for the disease (9, 10). Loss of *SMN1* and its translated product, the SMN protein, are the triggering events for spinal motor neuron loss and skeletal muscle atrophy in SMA. The identification of *SMN1* and a splice-defective paralog, *SMN2*, in the same locus raised the prospect of therapeutic intervention for SMA. Roughly two decades later, the first of three disease-modifying SMN-repletion treatments received regulatory approval for clinical use (11). By most measures — and relative to therapy development for similar rare diseases, such as amyotrophic lateral sclerosis (ALS) (12) and Duchenne muscular dystrophy (DMD) (13) — the studies that led to SMN repletion treatments have progressed quickly and yielded effective therapies. Yet it is clear that the current SMA treatments are limited in their effects (14, 15). Therapeutic outcome is critically dependent on the timing of intervention, the extent to which SMN can be restored to healthful levels, the precise levels to which SMN must be augmented in various organ systems to sustain their cellular activities, and the efficiency with which different organs are targeted by therapeutic agents. This review centers on therapeutic outcome linked to the tissue-specific requirements for SMN, focusing on one particular tissue — skeletal muscle — and how we think it contributes to disease pathogenesis. We begin with a critical examination of the historical evidence for an independent role for skeletal muscle in driving the neuromuscular dysfunction characteristic of SMA. We follow with a description of the effects of SMN paucity on mature myofibers and their progenitors, satellite cells, and include a discourse on molecular pathways potentially gone awry in these cells. We conclude with a discussion of possible outcomes of failing to restore SMN adequately to muscle and consider strategies that might be combined with currently approved treatments to ensure optimal muscle function. At a time when SMN repletion treatments have clearly altered the course of SMA, this Review is meant to refocus attention on one of several persisting challenges for the community of scientists, caregivers, and patients that may be encapsulated in the following related questions: (i) Does SMN paucity in muscle contribute significantly to the SMA phenotype? (ii) What are the mechanisms linking SMN to healthy muscle? (iii) How might one overcome deficiencies in currently available SMA treatments to ensure optimal therapeutic outcomes for muscle and thus the patient?

## Evidence of a role for muscle in SMA

SMA is commonly referred to as a spinal motor neuron disease (16). Consequently, muscle atrophy in the disease is often presumed to derive primarily from spinal motor neuron degeneration and denervation. Yet for clinicians and scientists familiar with the disease, and notwithstanding the neurodegenerative aspect of the condition, there has always been suspicion of a primary muscle defect in SMA. Indeed, such speculation dates as far back as the 1950s, when mild SMA was initially described (5). Patients with this relatively benign form of SMA were initially considered to have muscular dystrophy of the limb-girdle type with, among features associated with the latter condition, hypertrophy of the calf muscles. Other notable reports implicating a primary muscular component in SMA include one published in 1980 by Dutch scientists describing hypertrophy of calf muscles in patients with mild SMA that was accompanied by abnormally high levels of serum creatine kinase (CK) (17) — a marker of muscle breakdown. A study in the 1970s identified muscle pathology, including disorganization of the myofibrils, sarcomeres, and filaments, in patients with severe as well as mild SMA (18). More recently, rare instances of individuals with homozygous *SMN1* deletions have been reported to exhibit myopathic phenotypes rather than the classical neurogenic abnormalities associated with SMA (19, 20). While these more recent studies benefited from genetic confirmation of a diagnosis of SMA, the older studies described above were unable to rely on tests of *SMN1* integrity. Instead, SMA diagnoses were based on biopsies, overt phenotypes, and clinical presentation.

With the identification of the genetic cause of SMA in 1995, studies investigating a role for skeletal muscle in driving the neuromuscular SMA phenotype have become more refined. Nevertheless, those that relied on human muscle biopsies obtained prior to the identification of the *SMN1* gene may not, in every instance, have genotyped patient samples before utilizing them for investigation. Still, the experimental outcomes of these studies are thought-provoking and largely support a primary muscle defect in SMA. Notable among these are several that employed nerve and muscle cocultures to ascertain the disease-triggering effects of SMA muscle. These experiments revealed that when cultured with rat embryonic spinal cord explants, muscle cells from type I and type II, but not type III, SMA patients triggered rapid degeneration of the cocultures (21, 22). Interestingly, such degeneration was not observed when muscle cells from patients with distinct neurodegenerative diseases (ALS) or myopathies (DMD, nemaline myopathy, mitochondrial myopathies) were employed and, furthermore, did not appear to involve a soluble neurotrophic or neurotoxic factor (23).

Follow-up cell culture studies from the same group detected intrinsic defects of myogenesis in type I SMA. These defects, characterized by impaired fusion of type I SMA myoblasts into myotubes, were not observed with cells from patients with intermediate or mild SMA (24). Moreover, myogenesis defects occurred despite normal myoblast proliferation but were accompanied by reduced levels of nicotinic acetylcholine receptors in myotubes.

Notions of a discernible disease-causing effect of low SMN in muscle have been bolstered, reiterated, and further refined in the last two decades with the use of cultured cells, invertebrate models, and numerous independent lines of model mice. Several studies are worth highlighting. For instance, consistent with the existence of myoblast fusion defects in type I SMA patients, two studies — one employing C2C12 myoblast cells and one using satellite cells from a mouse model of severe SMA — reported perturbed myogenesis as assessed by quantification of multinucleate myotube formation (25, 26). In each instance, the muscle cells were purified and cultured without CNS tissue, diminishing any confounding influence of SMA motor neurons. These studies were accompanied by evaluation of myogenic factor expression in purified myoblasts and intact muscle from SMA model mice (26–28). The studies concluded unanimously that myogenic factor expression is perturbed under conditions of low SMN. However, the pattern of disrupted myogenic factor expression differed somewhat among the various studies. Whereas one (28) reported reduced Pax7, MyoD, and myogenin expression in muscle from symptomatic mutants of a commonly employed SMA mouse model that expresses two *SMN2* copies in an *Smn-*null background (29), another employing SV40 large T antigen–transformed myoblasts from the same line of mice demonstrated reduced Pax7 expression accompanied by an increase in MyoD and myogenin (27). Interestingly, muscle tissue extracted from a second, symptomatic intermediate SMA mouse model exhibited markedly elevated expression of all three myogenic proteins (28).

Notwithstanding these somewhat disparate findings, investigators explored how low SMN disrupts myogenesis. In the first of two elegant studies, it was shown that the myogenic program is activated prematurely in the most severe form of SMA but subsequently stalls as myofiber formation is initiated (26). In the second, the molecular basis of myoblast fusion defects was investigated and led to the discovery that two fusogenic factors — myomaker and myomixer — are reduced in severe SMA model mice and in C2C12 cells exhibiting modest (~55%) knockdown of SMN (30). Restoration of SMN in C2C12 myoblasts raised myomaker levels. Intriguingly, however, myomixer levels remained unchanged, and myogenic programming was not fully rescued. Still, AAV9-mediated overexpression of myomixer mitigated disease in a mouse model of SMA, suggesting that this factor links SMN to muscle pathology in SMA.

**Figure 1:**
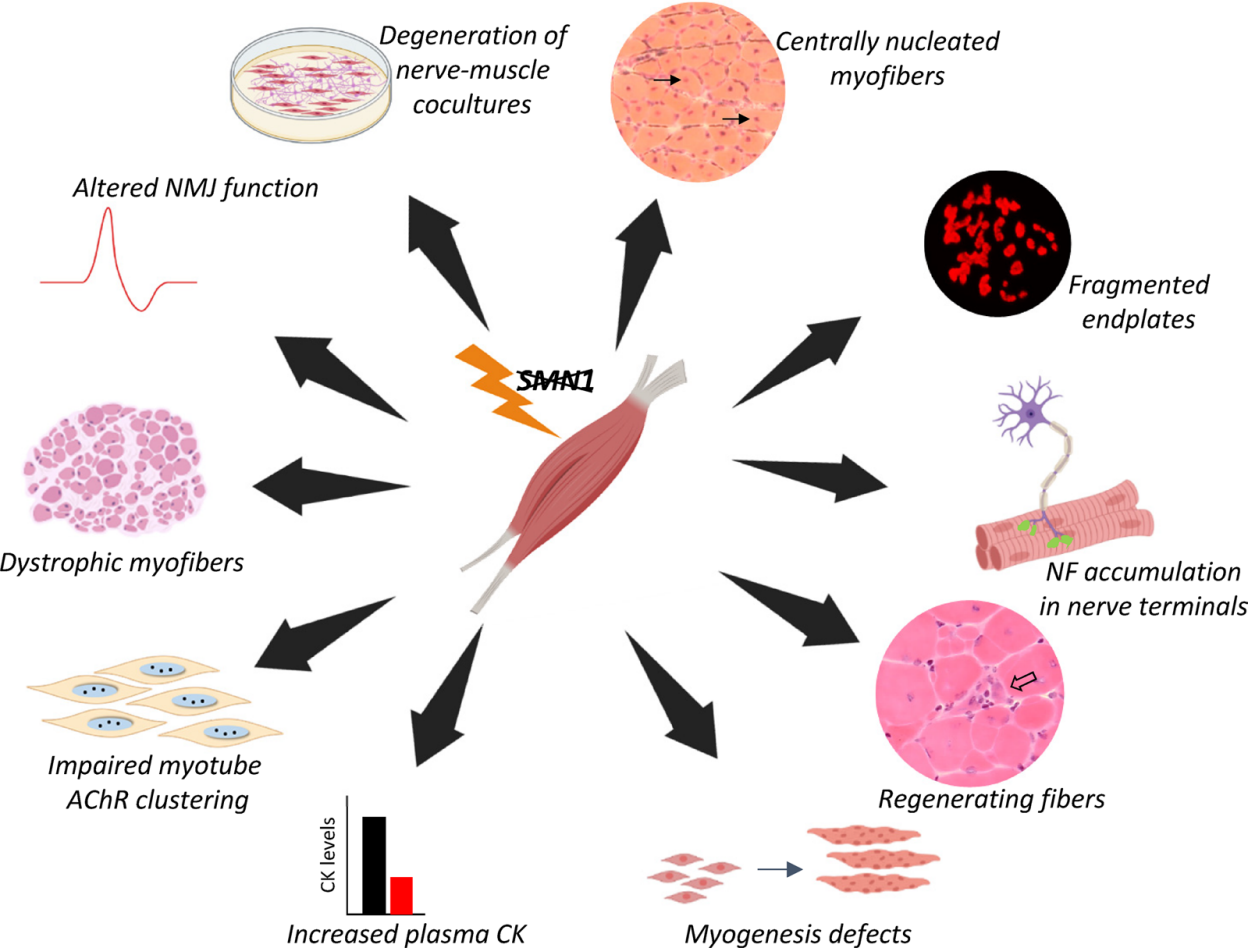
Schematic of the chief cell- and non-cell-autonomous defects arising from low SMN in skeletal muscle tissue. The loss of *SMN1* specifically in muscle results in both cell- and non-cell-autonomous effects. In muscle, reduced SMN levels leads to central nucleation of myofibers, altered regeneration, dysfunctional myogenesis, myofiber dystrophy, and impaired acetylcholine receptor (AChR) clustering. In addition, loss of SMN in muscle results in fragmented endplates, neurofilament (NF) accumulation at nerve terminals, increased circulating levels of creatine kinase (CK), altered function of neuromuscular junctions (NMJs), and nerve and muscle degeneration. Figure panels were constructed by the authors from material either generated in the laboratory or created using BioRender.com.

Additional evidence in support of a primary myopathy in SMA stems from investigations in invertebrate and mammalian models of the disease. In a fly model of SMA, morphology of the thoracic muscles was profoundly altered, and the authors of this study went on to show that SMN interacts and colocalizes with myofibrillar α-actinin (31). Consistent with the observation in flies, SMN was also shown to appear in perfect register with mouse myofibrillar α-actinin, suggesting a muscle-specific function for SMN protein that is conserved across species. In another study, mice with selective ablation of muscle SMN were reported to develop a severe dystrophic phenotype and died from the disease by approximately 1 month of age (32). While complete SMN ablation is incompatible with the survival of any cell type and does not accurately model human SMA, a more recent study (33) in which SMN was reduced to disease-relevant levels in skeletal muscle also concluded that low protein in this tissue is sufficient to trigger disease. Congruent with these findings, selective restoration of SMN to skeletal muscle of SMA mice restored myofiber size, increased animal weight, improved motor performance, and significantly enhanced life span relative to that of mutants ubiquitously depleted of SMN. Importantly, mice with skeletal muscle SMN restoration continued to exhibit neurodegeneration and loss of synaptic integrity due to persistently low SMN expression in the CNS (34). While these studies support an important role for skeletal muscle in driving the overall neuromuscular SMA phenotype, one report concluded otherwise (35). In this study, a *Myf-Cre* driver was used to deplete SMN in muscle but did not adversely affect the health of the resulting mice when assessed as young adults. Moreover, in contrast to the above-mentioned study by Martinez et al. (34), a study by Gavrilina et al. reported that selective restoration of SMN to myofibers of severely affected SMA mice failed to mitigate disease (36). The most salient of the various studies cited here are listed along with their main conclusions in Table 1. The chief pathologies associated with low SMN in skeletal muscle are further summarized and rendered as a schematic (Figure 1).

## Is SMA myopathy exacerbated by defective muscle progenitors?

Considering the evidence for a cell-autonomous role for skeletal muscle in driving SMA pathology, an obvious question centers on whether the myopathy originates in mature myofibers, muscle progenitors, or independently in the two cell types. In historical studies involving human SMA muscle biopsies, defects of the myofibers were the focus of attention and markedly easier to appreciate than possible defects of muscle satellite cells (18). On the other hand, outcomes of in vitro studies that employed myoblasts to detect defects of myogenesis imply defects originating in muscle progenitors (24, 27, 37). Attempts to discern the precise contribution of myofibers versus muscle progenitors to muscle defects in SMA were most directly initiated early this century (32, 38). In these studies, muscle-specific ablation of SMN, in mutants harboring two intact inducible *Smn*-knockout alleles floxed at exon 7 (*Smn^F7/F7^*), resulted in a discernibly milder phenotype than that observed in related mutants heterozygous for the intact allele (*Smn*^F7/*Δ*7^). The milder phenotypes of the *Smn^F7/F7^* mutants, which also harbored a human skeletal actin–Cre (HSA-Cre) driver to inactivate the floxed allele specifically in myofibers, were suggested to be the result of healthy satellite cells, which should have two intact alleles and therefore express WT levels of the SMN protein (38). Satellite cells expressing half the WT level of SMN were presumed to be the reason for the severe phenotype exhibited by *Smn*^F7/*Δ*7^ mice.

However, a more prosaic explanation for the disparate severities that was not considered is the inherent inefficiency of Cre-mediated inactivation of floxed alleles, and the possibility that mutants with two *Smn^F7^* alleles merely ended up harboring greater numbers of myonuclei with incomplete allele inactivation. As a result, such mutants would express incrementally higher levels of muscle SMN than *Smn*^F7/*Δ*7^ mutants, in which inactivation of just one intact floxed allele is required for total muscle SMN ablation.

Notwithstanding the caveat identified in the above study — and the debatable strategy of addressing the tissue-specific requirements for SMN by completely ablating rather than reducing SMN, as in human SMA — subsequent investigations reaffirmed the idea that defective satellite cells contribute to muscle pathology in SMA. Thus, in a mouse model that more accurately mimicked the genetics of human SMA, neonatal mutants had an average number of satellite cells exhibiting normal proliferative potential. However, differentiation of the SMA satellite cells was abnormal, based on the premature expression of myogenic markers, and these cells failed to generate myotubes efficiently (26).

**Figure 2:**
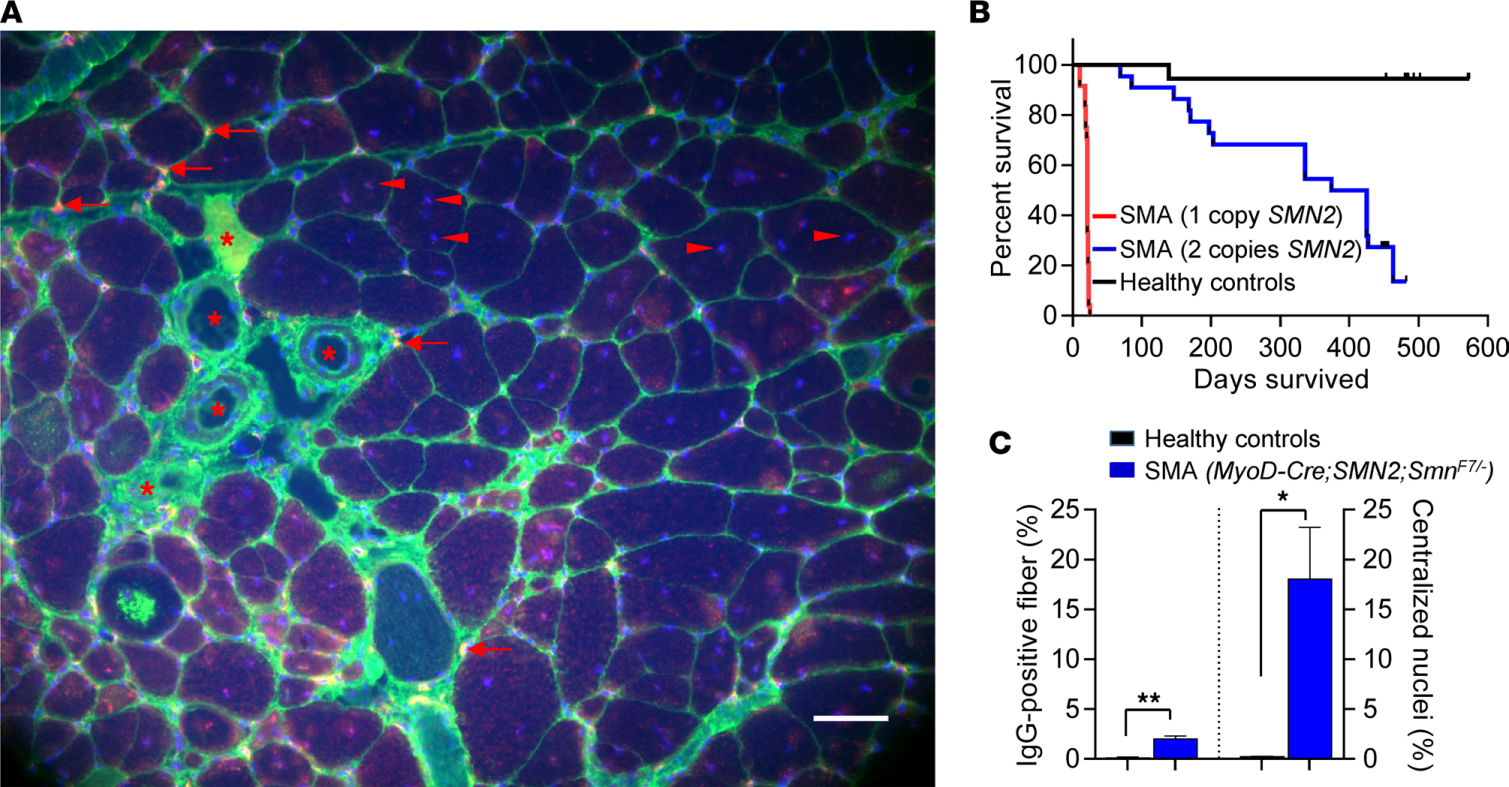
Depletion of SMN specifically in skeletal muscle is sufficient to cause pathology. (**A**) Confocal transverse section image of the calf muscle of a mutant mouse selectively depleted of the SMN protein in skeletal muscle tissue. Muscle cell–autonomous pathology is observed in the form of degenerating fibers penetrated by circulating IgG (asterisks), infiltrating microglia (arrows) and numerous myofibers containing abnormal, centrally positioned nuclei (arrowheads). Muscle was dual stained with antibodies against Iba-1 and mouse IgG to visualize microglia and damaged myofibers, respectively. Scale bar: 50 μm. (**B**) Kaplan-Meier survival curves depicting the correlation between *SMN2* copies, and thus absolute SMN levels, in muscle and life span of the SMA mutants. *P* < 0.0001, log-rank test, *n* ≥ 16 mice of each cohort. (**C**) Enumeration of degenerating myofibers and cells harboring central nuclei in the gastrocnemius of mutants selectively depleted of SMN in skeletal muscle. Roughly nine times as many SMA fibers were found to display central nuclei compared with those that were degenerating (IgG-positive). *P < 0.05, ** *P* < 0.01, *t* tests, *n* ≥ 300 fibers from *n* ≥ 3 mice of each cohort. Panels adapted from Kim et al. (33).

Novel lines of model mice were generated to cement notions of a disease-triggering role for low SMN in skeletal muscle and address questions of the origin of muscle cell–autonomous defects in SMA more accurately (33). Two distinguishing characteristics made these mutants especially useful. First, SMN depletion was specifically targeted to skeletal muscle; and second, low SMN in muscle was nevertheless maintained, as in human SMA, by expression of one or two copies of *SMN2*. Accordingly, the resulting mutants not only expressed disease-relevant levels of SMN in muscle but also enabled investigation of the effects of depleting the protein in both muscle progenitors and mature myofibers. Several inferences may be drawn from these analyses. First, depletion of SMN in skeletal muscle progenitors is sufficient to trigger disease (Figure 2A). Moreover, the severity of the myopathies and overall phenotypes closely correlated with absolute SMN levels (Figure 2B). Second, examinations of mutants that derived residual muscle SMN from two *SMN2* copies hinted at a role for this protein in satellite cells, implying that myopathy in SMA must originate, at least in part, in these progenitors. For instance, it was shown that compared with the modest muscle degeneration observed in mutant animals, a disproportionately large number of myofibers had centrally located nuclei (Figure 2C). Such nuclei are generally associated with regenerating fibers but may also be a consequence of untimely activation — consistent with premature differentiation of these cells (26). Finally, restricting SMN depletion to mature myofibers instead of inducing it in muscle progenitors resulted in milder phenotypes. Still, the eventual appearance of muscle pathology in this last set of mutant mice (*HSA-Cre;SMN2;Smn^F7/–^)* unequivocally assigned a role to SMN in sustaining the health of mature myofibers. Collectively, the various studies cited here suggest that low SMN is damaging to both muscle progenitors and the myofibers that arise from them.

## How does SMN maintain muscle health and function?

If the evidence cited here, which in our view is compelling, is truly reflective of a cell-autonomous role for muscle in driving SMA pathology, it prompts several questions. First, how does SMN maintain the health of myofibers and their progenitors? Second, what are the minimum levels of SMN required by muscle to sustain viability? Third, is the level of SMN necessary to ensure early postnatal muscle growth identical to that required for muscle maintenance? Fourth, do muscles, akin to motor neurons, exhibit differential vulnerabilities to low SMN? Finally, what are the potential repercussions of failing to restore muscle SMN to healthy levels and how can muscle SMN deficiency despite treatment with SMN augmenting agents be overcome? In the remainder of the Review, we attempt to address these biologically and clinically relevant questions.

SMN has been implicated in a multitude of functions (39) and new ones continue to be revealed (40). Yet there is no obvious function, pathway, or set of factor(s) that connect SMN uniquely to the health of muscle. One intuitive means of establishing links between SMN and muscle health involves a careful multiomic analysis of SMA and healthy muscle. In this regard, the newer lines of SMA model mice — which not only vary in severity, but are engineered to preclude the confounding influence of low SMN in neighboring tissue — could be especially useful (33). For example, comparisons of perturbations in mice with muscle-specific SMN depletion to those in mutants with systemically low SMN ought to be instructive and distinguish primary muscle defects from those downstream of motor neuron dysfunction. We advocate for such investigations to be carried out not only on different muscles from the model mice but also — to the extent possible — on patient tissue. Specifically examining muscle progenitors in this manner is also certain to cast novel light on myopathy in SMA. The evidence for a role for SMN in satellite cells is persuasive, yet not unequivocal. These cells are critical not only during early postnatal muscle growth (41) but also as a means of replenishing injured or aged muscle. Indeed, the importance of these cells has been described in several muscle diseases, notably DMD, limb-girdle muscular dystrophy, myopathy associated with dystroglycan dysfunction, Fukuyama congenital muscular dystrophy, and sarcopenia (42–46). Moreover, investigations of the cells in the context of these conditions have cast important light on their characteristics, how they respond to build or replenish lost muscle, whether their activities ameliorate or worsen disease, and the mechanisms that operate within them to balance self-renewal with differentiation (47). These studies have revealed that only a small (10%–20%) proportion of satellite cells, defined by their expression of the transcription factor Pax7, are truly quiescent and able to self-renew. Such self-renewal is critically dependent on the capacity for asymmetric cell division — into a quiescent satellite cell and a second activated myoblast that initiates the myogenic program. The second cell, which continues to express Pax7 in the short term and is therefore frequently attributed the status of a muscle stem cell, engages in symmetric cell division to expand the number of activated myoblasts and, consequently, grow or replace lost muscle. A fine balance exists between asymmetric and symmetric satellite cell division, and one that is regulated by a complex network of factors including signaling from Notch, Jak2/Stat3, Wnt7a, and p38MAPK (48–56). Disruption of this balance exacerbates muscle loss in a variety of myopathies (57). Indeed, gradual satellite cell exhaustion and eventual senescence, owing to protracted cycles of myofiber degeneration and regeneration, in DMD (58, 59) are aggravated by loss of muscle protein in these cells. Dystrophin, for instance, establishes satellite stem cell polarity and thus regulates asymmetric division into quiescent and activated daughter cells (60). Could SMN also influence such pathways, either through orchestration of the splicing cascade and subsequent expression of factors important to muscle function or in some hitherto undescribed manner? Investigation of such questions, particularly in light of reports of premature satellite cell activation in SMA, is a biological and clinical imperative.

Despite a meager understanding of pathways underlying a specific role for SMN in muscle satellite cells, there are some aspects of SMA myopathy that are not only well recognized but also explained by phenomena revealed in other muscular dystrophies. For instance, studies of model mice suggest that levels of SMN typical of type I SMA will likely have a profound and direct effect on muscle (33). In what resembles an all-or-none effect, an incremental increase in the protein significantly mitigates disease severity and muscle pathology.

Thus, model mice expressing SMN from two rather than one *SMN2* copy are relatively mildly affected. Early postnatal muscle development is restored, and life span is extended from roughly 2½ weeks in the former mutants to approximately 13 months in the latter. Additionally, there is little evidence of perturbed myogenic factor expression in the mutants with the mild phenotype, and onset of muscle pathology in these mice is considerably delayed. This phenotype suggests, at least in model mice, that two *SMN2* copies are sufficient to ensure muscle growth through prepubertal life but fail to maintain myofiber health during adulthood. Moreover, certain muscles, such as the flexor digitorum brevis (FDB), are more vulnerable to low SMN than others (33), with myopathy, in general, likely to be accelerated by sustained muscle activity. Elevated CK values, especially in patients with milder SMA, are suggestive of delayed-onset, activity-driven myopathy (61). Still, studies to better explain how low SMN triggers muscle pathology are urgently needed and not only promise to shed new light on SMN biology but could also prove useful in developing and optimizing novel SMA therapies for patients. Such studies will also be instructive for understanding the differential vulnerability of muscles to low SMN (62, 63) and the extent to which the vulnerability arises in the pre- versus postsynaptic compartments of neuromuscular synapses. Low SMN in muscle appears to have a retrograde effect on nerve terminals, as suggested by evidence of neurofilament accumulation in the terminals of severely affected mutants with skeletal muscle–specific depletion of SMN (33). Whether this pathology eventually results in denervation of the muscle remains to be determined and will have to be assessed in milder mutants with life spans long enough for the process of axonal retraction to run its course. Nevertheless, it is clear that neuromuscular junction (NMJ) function is altered in mice selectively depleted of SMN in muscle, as quantal content, a measure of neurotransmission is perturbed in these mutants (33).

## Conclusions and the path ahead

Although low SMN in skeletal muscle is sufficient to damage the tissue and very likely triggers defects not just in myofibers but in muscle stem cells as well, the molecular mechanisms underlying the pathology remain poorly understood. Defining these mechanisms is important not only because SMN, like other muscle proteins such as dystrophin, sustains muscle health, but also because doing so informs treatments for SMA. Nusinersen, the antisense oligonucleotide approved for treatment of the disease, is administered intrathecally and does not increase levels of intact SMN transcript from *SMN2* in muscle of treated patients (64).

Onasemnogene abeparvovec, an AAV9-mediated gene replacement therapy for SMA, is delivered systemically and therefore expected to target skeletal muscle. However, treatment with this agent is not without caveats. For instance, it is currently only approved for patients under two years of age. Moreover, as it is a one-time treatment, muscle turnover in treated patients would eventually result in loss of the episomal therapeutic molecule and reversion of transduced cells to an SMA state. Finally, it is unclear whether AAV9 delivers the replacement gene efficiently to nondividing muscle stem cells. AAV6, a related capsid that exhibits robust tropism for muscle, fails to target satellite cells (65), and it is possible that AAV9 displays similar characteristics. Risdiplam — a small-molecule splice-switcher and the most recently approved SMN-augmenting agent to be added to the arsenal of SMA therapies — is reported to modulate *SMN2* splicing systemically but has only relatively modest effects on boosting the protein, raising plasma SMN levels in treated types I and II patients by approximately 2.5-fold (66, 67) and muscle SMN of mutant mice by 210% (68). Given the profoundly low (10%–15% of WT) baseline levels of SMN in severely affected patients (64, 69), treatment using currently approved doses of an agent such as risdiplam is unlikely to raise SMN to what are considered the “safe” levels found in asymptomatic carriers. Rather, protein may only be increased to levels (30%–40%) approximating those in patients with type III or type IV SMA (70, 71), which is insufficient to prevent disease. These observations, and empirical data about the requirements for SMN in muscle (33), raise the question: What sort of therapeutic outcome might one expect, particularly in the long term, from current SMA therapies? One potential outcome, notwithstanding an immediate benefit following treatment of the presymptomatic patient, is the evolution of a delayed-onset, chronic myopathy. Onset and severity of such pathology will almost certainly vary and be subject to SMA type, treatment timing, and targeting efficiency.

While the outcome described above is not inevitable, clinicians and families ought to be prepared; therefore, it is prudent to consider adjunct treatments to ensure that the health of muscle in treated patients is sustained. There are several strategies worth considering. One strategy that may be considered targets the TNF-like weak inducer of apoptosis (TWEAK)/TNF receptor superfamily member FGF-inducible 14 (Fn14) pathway. This pathway has been implicated in myriad cellular activities, including muscle protein degradation, atrophy, muscle oxidative metabolism, and myofiber regeneration (72). Upregulation of the pathway following muscle damage suggests that it may be involved in tissue repair. Indeed, in two mouse models of SMA, expression of TWEAK and Fn14 was reduced. Moreover, stimulating the pathway with an Fc-TWEAK agonist modestly mitigated disease in the two models (73). Selective androgen receptor modulators (SARMs) might also be considered in combating muscle dysfunction in SMA. These agents, which mimic the muscle-building anabolic effects of androgens without their pronounced adverse effects — including virilization in women and cardiac and prostatic hypertrophy — were initially developed for the treatment of sarcopenia but have also produced benefit in a rodent DMD model (74–76). The adjunct therapy that has perhaps garnered the greatest interest as a means to combat primary muscle disease involves interfering with myostatin signaling. Myostatin, a member of the TGF-β superfamily, is expressed predominantly in skeletal muscle, where it negatively regulates the growth of the muscle by binding to the type IIB activin receptor (ActRIIB) (77). Blocking myostatin activity is known to increase skeletal muscle mass (78, 79) and was reported to mitigate disease in the *mdx* mouse model of DMD (80). Accordingly, a number of studies have investigated the effects of suppressing myostatin activity for SMA (81–87). These preclinical studies have produced disparate data, with some claiming benefit and others failing to mitigate disease. Nevertheless, the positive outcomes reported have appeared promising enough to prompt a number of clinical trials (88–90). One of these, which employed apitegromab — a human monoclonal antibody that binds the pro-forms of myostatin, thereby inhibiting its activation — concluded that the agent, when used in combination with nusinersen, synergized with the SMN-augmenting therapeutic molecule to ameliorate disease (91). Despite the range of options presented here, it is unclear to what extent any of the associated pathways truly restore SMN activities or functions to muscle. Without restoring such activities, the various adjunct treatments considered here will be of incremental value. This sobering thought justifies renewed efforts to define precisely how SMN sustains muscle health. Identifying the molecular pathways and the various mediators that govern this process remains the most assured means by which any myopathy in SMA will eventually be addressed in the clinic.

## Figures and Tables

**Table 1:**
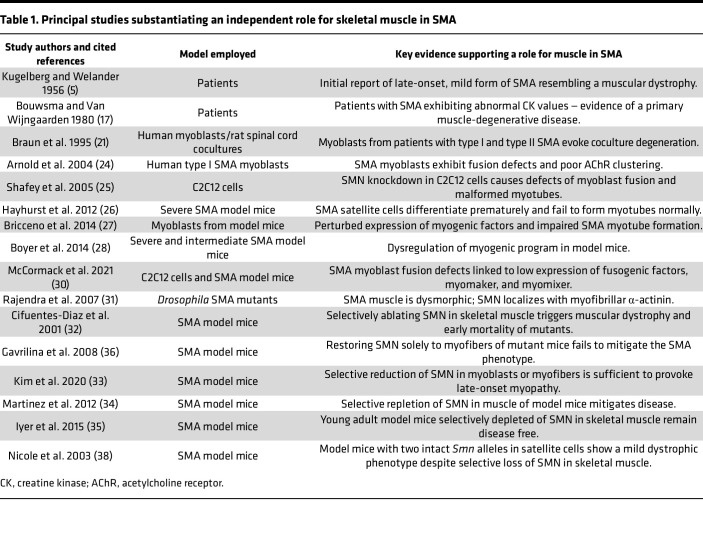
Principal studies substantiating an independent role for skeletal muscle in SMA

## Version 1 09/22/2023

Electronic publication

## Version 2 09/22/2023

The article title was corrected
